# Feasibility and validity of a single camera CNN driven musculoskeletal model for muscle force estimation during upper extremity strength exercises: Proof-of-concept

**DOI:** 10.3389/fspor.2022.994221

**Published:** 2022-09-23

**Authors:** Lisa Noteboom, Marco J. M. Hoozemans, H. E. J. Veeger, Frans C. T. Van Der Helm

**Affiliations:** ^1^Department of Human Movement Sciences, Faculty of Behavioural and Movement Sciences, Vrije Universiteit Amsterdam, Amsterdam, Netherlands; ^2^Department of Biomechanical Engineering, Faculty of Mechanical, Maritime and Materials Engineering, Delft University of Technology, Delft, Netherlands

**Keywords:** musculoskeletal modeling, strength training, fitness, markerless motion capture, artificial intelligence, video-based motion capture

## Abstract

Muscle force analysis can be essential for injury risk estimation and performance enhancement in sports like strength training. However, current methods to record muscle forces including electromyography or marker-based measurements combined with a musculoskeletal model are time-consuming and restrict the athlete's natural movement due to equipment attachment. Therefore, the feasibility and validity of a more applicable method, requiring only a single standard camera for the recordings, combined with a deep-learning model and musculoskeletal model is evaluated in the present study during upper-body strength exercises performed by five athletes. Comparison of muscle forces obtained by the single camera driven model against those obtained from a state-of-the art marker-based driven musculoskeletal model revealed strong to excellent correlations and reasonable RMSD's of 0.4–2.1% of the maximum force (Fmax) for prime movers, and weak to strong correlations with RMSD's of 0.4–0.7% Fmax for stabilizing and secondary muscles. In conclusion, a single camera deep-learning driven model is a feasible method for muscle force analysis in a strength training environment, and first validity results show reasonable accuracies, especially for prime mover muscle forces. However, it is evident that future research should investigate this method for a larger sample size and for multiple exercises.

## Introduction

Knowledge of the level of muscle forces during sports activities can be essential for injury risk estimation and performance enhancement. Especially in strength training, a sports domain that deals with a high prevalence of (mainly upper extremity) muscle injuries (1), athletes could benefit from knowledge of their personal muscle load during a workout, to optimize the training stimulus and prevent muscle overload. Multiple methods for muscle force estimation can be employed in human movement and sport science. A common method is surface electromyography (EMG), a technique that detects muscle activation signals from electrodes placed on the skin. Another method is to employ a musculoskeletal model, a computational representation of the human musculoskeletal system, to simulate movements based on captured motion and external force data (e.g., from dumbbells). *Via* a process called inverse dynamics, the net internal joint moments responsible for the given motions can be estimated by solving equations based on Newton's laws of motion (2). Subsequently, the model can estimate the most cost-effective combination of muscle forces required to deliver this net joint moment (2). Typically, motions for musculoskeletal modeling are captured by an optoelectronic measurement system (OMS) (state-of-the-art). An OMS consists of multiple cameras that detect light from either active or passive markers placed on an athlete's skin, usually at or related to predetermined bony landmarks, to determine the three-dimensional (3D) location of those markers by time-of-flight triangulation (3). Another option to capture motions for musculoskeletal modeling is by placing inertial measurements units (IMUs) on body segments of the athlete. IMUs consist of an accelerometer, gyroscope and usually a magnetometer and combine the data from these sensors to obtain segment orientations (3).

However, these described methods for muscle force estimation are not easily applicable on a large scale in a gym environment. For EMG, sensor placement can be time-consuming, estimating muscle force levels from muscle activations can be complex, and only the activation of superficial (large) muscles groups can be detected. For marker-based kinematic assessments, limitations include high costs, long set-up times, soft tissue artifacts, and the restrictions in performance because of the laboratory setting (3). Furthermore, positions obtained from IMU data can suffer from large integration drifts, and IMU sensors are sensitive to measurement errors when there is metal nearby, which could form a large issue in a gym environment (3). In addition, all these methods are accompanied by limitations in activity performance due to the attachment of equipment to participants.

A potential solution to make muscle force analysis applicable for the gym is by using markerless pose estimation in combination with a musculoskeletal model, as markerless measurements are advantageous in terms of costs, set-up time and not restricted to a laboratory setting. Markerless methods combine recordings from one or multiple standard or depth cameras with a computer vision algorithm to estimate 2D or 3D locations of joint centers (3). The Microsoft Kinect is a commonly used markerless pose estimation tool in biomechanics, which uses an infra-red depth sensor and a random forest approach to obtain 3D joint kinematics (4). Interestingly, the large field of pose estimation is developing rapidly and more sophisticated algorithms than used by the Kinect can be employed. With the rise of deep neural networks (DNNs), pose estimation accuracies improved substantially over the past years, with the best recent models reporting mean per joint position errors (MPJPE) of about 20 mm (5, 6). DNNs are well-suited for pose estimation because their multi-layer structure allows for incorporation of a lot of data to optimize estimations while remaining efficient (7, 8). For instance, temporal information of previous joint position solutions, and information of other joint positions can all be used in the estimation, whereas the random forest approach makes estimations separately for each joint and based on single images (7). DNNs could therefore potentially be more accurate and more robust in situations like temporary segment occlusion. Especially convolutional neural networks (CNNs), a type of DNNs, are powerful for image-processing and are typically employed for pose estimation (5). Remarkably, some of these networks can even estimate 3D joint positions from a single 2D video, which means that only a single standard camera is required for the recordings (8). When joint centers obtained from a single camera and CNN are used to drive a musculoskeletal model, this could result in a feasible method to obtain muscle forces in a sports environment.

However, as stated by Wade et al. (9), the transfer of deep learning-based pose estimation methods toward application in biomechanics has been slow, potentially due to the requirement of advanced coding skills and in-depth computer science knowledge. Indeed, only few studies have evaluated markerless driven musculoskeletal models so far (10–12), and all used the Kinect rather than a deep learning-based method. Moreover, from those studies only Skals et al. (10) assessed muscle forces as output parameter. Although promising root-mean-square-deviations (RMSDs) and correlations for the middle deltoid muscle forces compared to forces obtained from a marker-driven model were found during a loaded and unloaded lateral raise task (2.88–5.45% of body weight (BW) and r = 0.66–0.88, respectively), one muscle is not sufficient for validation of a complete upper-extremity musculoskeletal model given the complexity and coherence of the system. Therefore, the validity of muscle forces estimated by markerless musculoskeletal modeling remains to be determined. In addition, Skals et al. (10) used a set-up with two Kinect sensors, which still requires calibration that can be time-consuming. There is need for assessment of a more practical set-up, requiring only a single standard camera for the recordings.

Therefore, the objective of the present study was to assess the feasibility of a single camera, CNN driven musculoskeletal model (proof-of-concept) and provide some first clues regarding the accuracy of estimated upper-body muscle forces (including prime mover and stabilizing muscles) during strength exercises, by comparing against forces obtained from a (state-of-the-art) marker driven musculoskeletal model. To bridge the gap between deep-learning and biomechanics, an open source pretrained model that included a clear step-by-step documentation on GitHub was used. The presented method should therefore also be suited for biomechanical researchers or clinicians without extensive deep-learning knowledge.

## Materials and methods

### Participants

Five healthy male participants (mean ± SD age 16.8 ± 1.3 years, body mass 80.4 ± 4.2 kg, body height 1.84 ± 0.07 m) were included in this study. The participants were high-level baseball pitchers with a lot of experience in strength training. The study was approved by the local ethics committee of the Faculty of Behavioral and Movement Sciences, Vrije Universiteit Amsterdam (VCWE-2019-033). All participants provided written (parental) informed consent.

### Procedure

**Figure 1:**
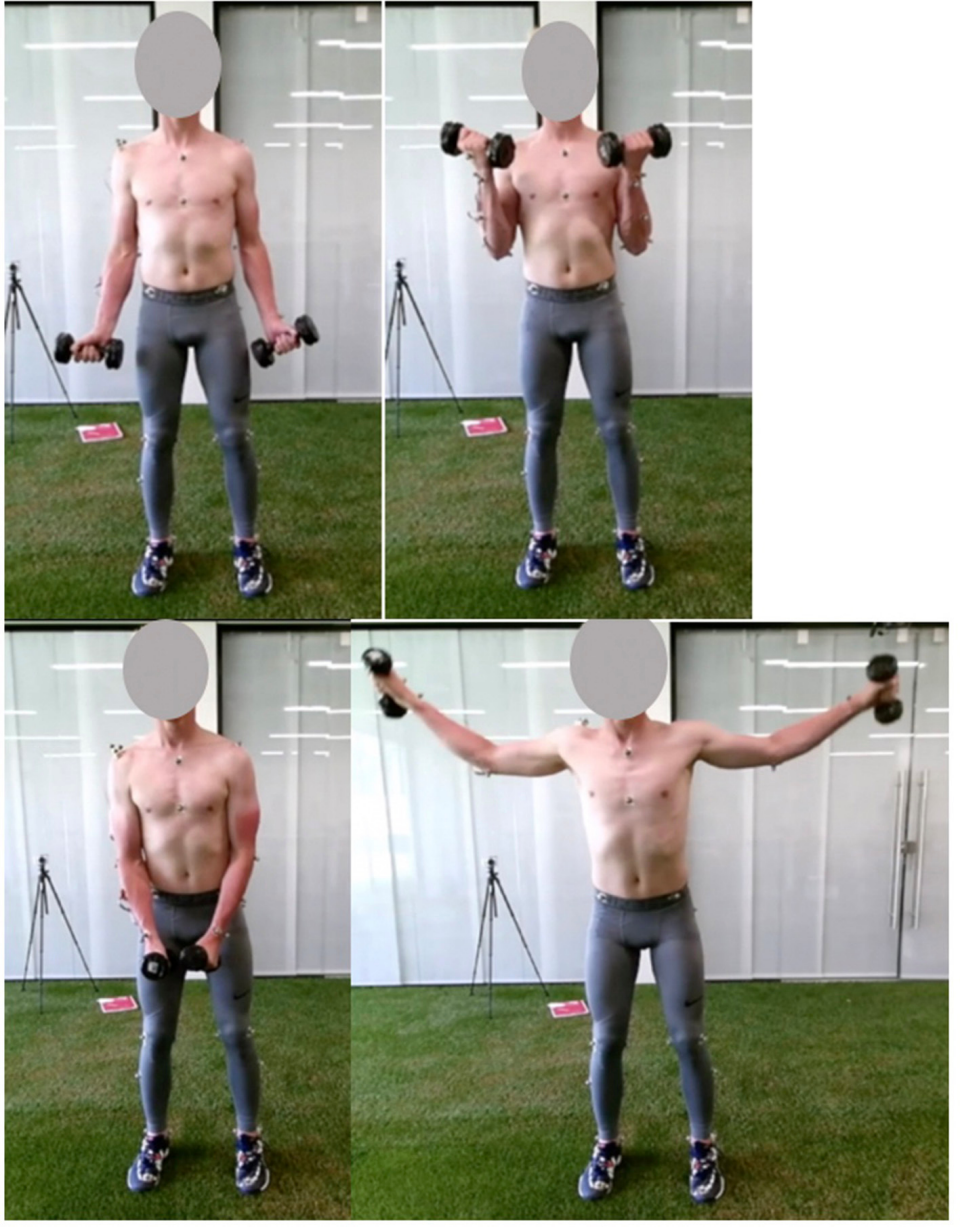
Pictures of the start (left) and mid (right) pose of the biceps curl (top) and lateral fly (bottom) exercise.

During preparation, participants were equipped with a set of 12 reflective markers on the thorax and dominant arm (Table 1). Subsequently, participants performed a 10-min warm-up protocol. During the actual measurements, participants performed two upper extremity dumbbell exercises: the lateral fly and the biceps curl (Figure 1). The lateral fly movement occurs predominantly in the frontal plane, whereas the biceps curl movement occurs mainly in the sagittal plane. In addition, axial rotation of the upper and lower arm can be expected during the lateral fly, and segment occlusion of the upper arm can be expected during the biceps curl, allowing for evaluation of the motion capture system and musculoskeletal model during these challenging conditions. In total, each exercise was performed for 3 sets of 5 repetitions, at a self-selected pace. In-between each set participants rested for 30 seconds. The mass of each dumbbell was 5 kg for the biceps curl and 3 kg for the lateral fly.

**Table 1:** Anatomical locations of markers/coordinates used per system.

**Segment**	**Marker-based**	**Camera-based**
	**method**	**method**
Thorax	Incisura Jugularis	Mid spine
	Processus Xiphoideus	Mid thorax
	Cervical Vertebrae 7	
	Thoracic Vertebrae 10	
Upper arm (dominant side)	Acromion	Shoulder joint center
	Epicondylus Medialis	
	Epicondylus Lateralis	
	Upper arm (tracking marker)	
Lower arm (dominant side)	Head of the Ulna	Elbow joint center
	Styloid processes of radius	
	Lower arm (tracking marker)	
Hand (dominant side)	Interphalangealis proximal III	Wrist joint center

### Materials

**Figure 2:**
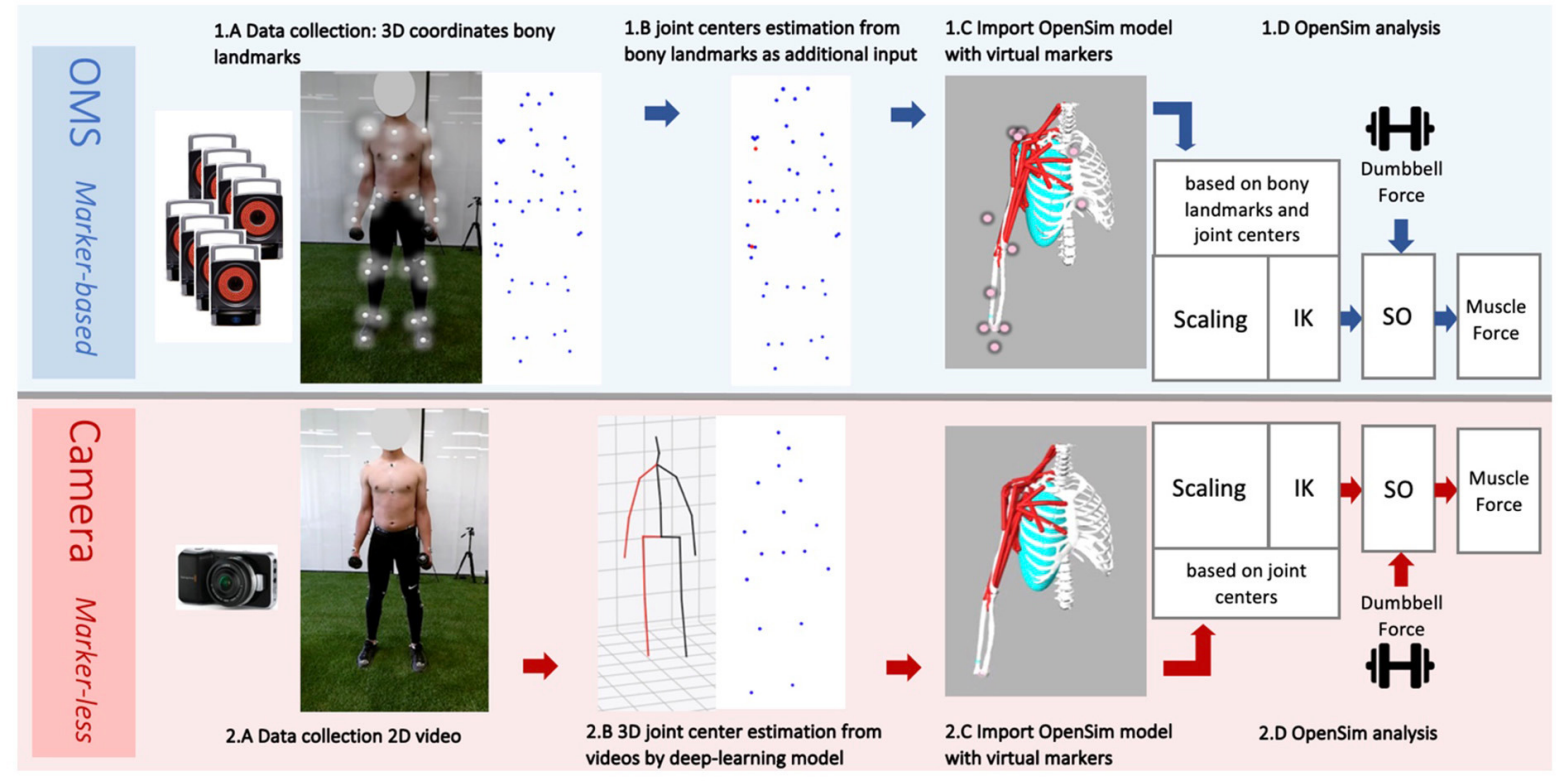
Workflow data collection and analysis from the marker-based optolectronic measurement system (OMS) (top) and the markerless camera (bottom) methods. The OMS consisted of 8 infrared cameras that captured the 3D trajectories of reflective markers placed on bony landmarks of the participant **(1.A)**. Additionally, joint center trajectories were estimated from bony landmarks and added to the data **(1.B)**, before importing in OpenSim. The generic thoracoscapular model with virtual markers (pink) that corresponded to locations of the collected bony landmarks and joint centers (experimental markers) (blue and red) was imported in OpenSim **(1.C)**. The camera captured standard 2D videos of the participant from a frontal view **(2.A)**. A deep-learning model (8) was employed to obtain 3D joint center trajectories from these videos **(2.B)**, which were subsequently imported into OpenSim. The generic thoracoscapular model was also imported in OpenSim with virtual markers (pink) (difficult to see in the figure as these are located within the joints) that corresponded to locations of the joint centers (experimental markers) (blue) **(2.C)**. The OpenSim pipeline was almost the same for both methods **(1.D and 2.D)**, including the steps: scaling, inverse kinematics (IK) and static optimization (SO) (with the external dumbbell force as additional input). The only difference was that for scaling and IK, experimental markers from bony landmarks and joint centers were used for the marker-based method, whereas only joint centers were used for the marker-less camera method.

The whole data collection and processing pipeline is presented in Figure 2. The performed strength exercises were simultaneously measured by two motion capture systems: an opto-electrical measurement system (OMS) (Figure 2 1.A) and a normal RGB camera (Figure 2 2.A) built-in in a Kinect (v2, Microsoft Corporation, Redmond, WA, USA) (depth sensor not used) which was used for the single camera markerless motion capture. The OMS (Vicon Motion Systems, Oxford, United Kingdom) consisted of eight high-speed infrared cameras that registered the 3D coordinates of the reflective markers at a sample frequency of 400 Hz. Coordinates were expressed in the laboratory's coordinate system and recordings were processed with the Vicon Nexus software (Vicon Motion Systems, Oxford, United Kingdom). The single camera was placed 1.5 meters in front of the subject at a height of 1.5 meters and a downwards angle of 15 degrees. The sample frequency of the camera was 30 Hz and the image resolution was 1,920 x 1,080 pixels.

### Pose estimation model

To generate 3D joint center coordinates from the captured videos, a two-step approach for 3D pose estimation, developed by Pavllo et al. (8), was used (Figure 2 2.B). In their approach, first a pretrained convolutional neural network (CNN) is used to detect a person in an image, and detect their 2D joint center locations, which are subsequently lifted to 3D using a newly developed temporal dilated convolutional model. The second step additionally allows for modeling of temporal relations between individual poses. A recent review (6) revealed that this model from Pavllo et al. (8) is one of the most accurate single-view models with mean per joint position errors (MPJPE) of 23.1 mm on the HumanEva dataset and 46.8 mm on the human 3.6M dataset. This model is available from an open-source well-documented GitHub repository (13), making this method readily available and easy to use without requiring extensive deep-learning knowledge. To apply this model on the recorded videos, the steps under (14) were followed. A Detectron2 model trained on the COCO dataset was used for 2D keypoint detection, and the pretrained h36m detectron coco.bin model was used for the final 3D pose estimation, which was pretrained on the extensive Human 3.6M dataset. This method generated the 3D coordinates of 17 joint centers for the full body, from which five were used as input for the upper-body musculoskeletal model (Table 1).

### Data cleaning and preparation

For the eventual analysis, the program OpenSim will be used. OpenSim is free, open-source software that allows users to run simulations with computational models of the human musculoskeletal system. By simulating recorded motions with an OpenSim model in combination with external forces (e.g., forces from dumbbells) it can be estimated which muscle forces are required to obtain the given motions and external forces by following the steps: inverse kinematics (IK), inverse dynamics (ID) and static optimization (SO) (these steps are elaborated in the following paragraphs). The motion data from the marker-based and from the deep learning-based method had to be cleaned and prepared before it could be processed in OpenSim. Coordinate data from the marker-based method were first imported in MATLAB (2020a, The MathWorks, Inc., Natick, Massachusetts, United States) to correct the data for switched or missing markers, under the hypothesis of rigid bodies. Some trials had to be excluded due to too many missing markers (minimal three markers required per segment).

Subsequently, joint center locations of the shoulder, elbow, and wrist, were estimated from bony landmarks (Figure 2 1.B). This step is recommended by the guidelines of OpenSim, as the tracking accuracy of the model likely improves when joint center trajectories are used in addition to the bony landmark trajectories. For the shoulder, the joint center was calculated based on regression equations reported by De Leva (15). For the elbow and wrist, the joint centers were calculated as the midpoint between the medial and lateral epicondyle markers, and the midpoint between the radial and ulnar styloid markers, respectively (16). For the coordinate data generated by the deep-learning model, visualizations of the joint center stick figures (that were provided along with the coordinate data), were inspected to check if estimations seemed realistic. In the case of completely insufficient and unrealistic joint tracking, trials were excluded. No further data post-processing or cleaning was required for the single camera deep learning-based method.

### Musculoskeletal model

**Figure 3:**
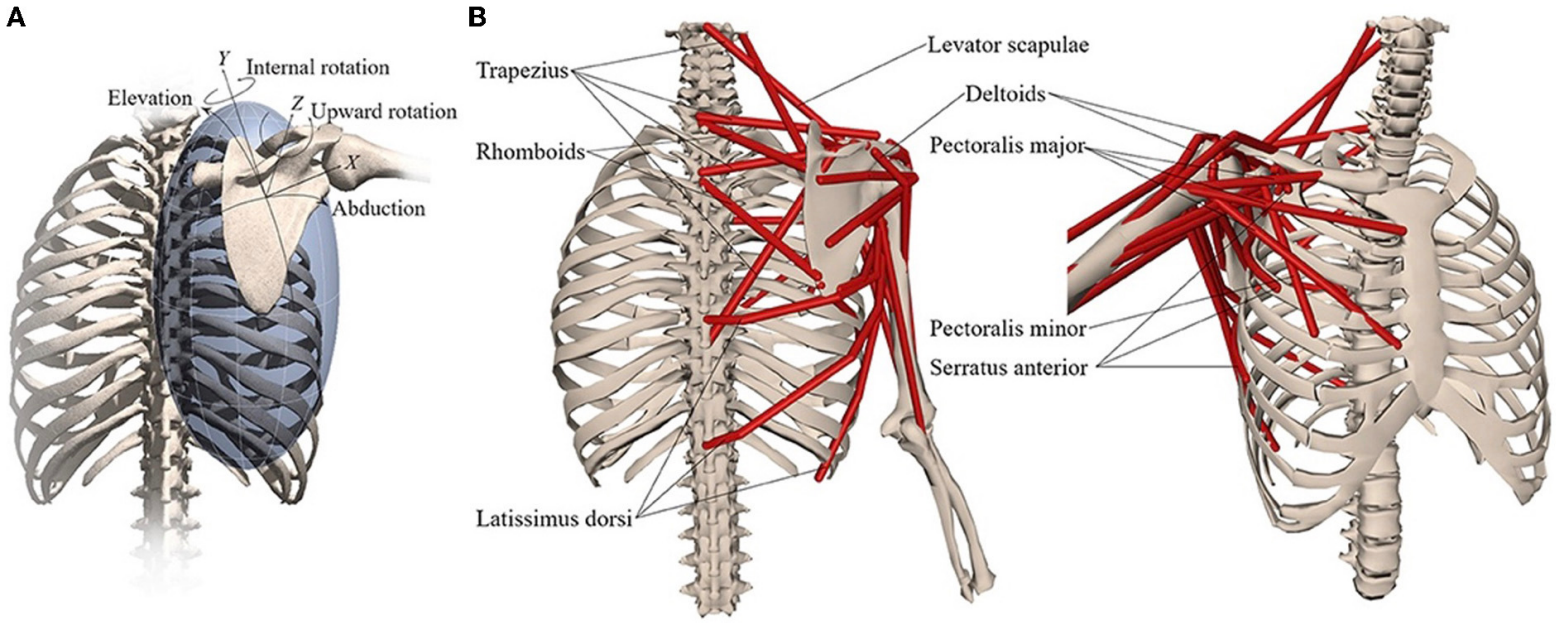
Musculoskeletal model with **(A)** scapular degrees-of-freedom and **(B)** shoulder muscles that control the scapula. Reprinted from Muscle contributions to upper-extremity movement and work from a musculoskeletal model of the human shoulder by Seth et al. (17).

An OpenSim musculoskeletal model of the shoulder and elbow (Figure 3) (17) was used in the present study. The model included muscle parameters and architecture based on Breteler et al. (18), with aggregated muscle bundles from Van der Helm (19) (Table 2), combined with an accurate model of scapulothoracic kinematics (20). Two generic models were used, one for the camera-based input data and one for the marker-based input data. The marker-based model contained so-called virtual markers (pink markers in Figure 2 1.C) on locations that corresponded to the experimental markers from bony landmarks and joint centers (blue and red markers in Figure 2 1.B), whereas the camera-based model only contained virtual markers at the joint centers (which cannot be seen clearly in Figure 2 2.C because these virtual markers are within the joints).

**Table 2:** Thoracoscapular shoulder model muscle parameters adapted from Breteler et al. (18) with aggregated bundles from by Van der Helm (19).

**Muscle**	**Group**	**Max isometric force**	**Optimal fiber length**	**Tendon slack length**	**Pennation angle**	**Van der Helm bundles**
Trapezius	Scapula superior	1,043	0.1127	0.027	0	1–6
	Scapula middle	470.4	0.0832	0.032	0	7–9
	Scapula inferior	414.4	0.1264	0.035	0	10–12
	Clavicle	201.6	0.1116	0.027	0	C1–C2
Serratus anterior	Superior	387.8	0.0945	0.000	0	9–12
	Middle	508	0.1538	0.012	0	5–8
	Inferior	430	0.1587	0.000	0	1–4
Rhomboideus	Superior	200.2	0.0986	0.015	0	1–2
	Inferior	407.4	0.1152	0.028	0	3–4
Levator scapulae		280	0.1578	0.019	0	All
Coracobrachialis		648.2	0.0683	0.104	0	All
Deltoideus	Anterior	707.7	0.0940	0.088	5	C1–C4
	Middle	2,597.8	0.0748	0.064	5	4–11
	Posterior	1,324.4	0.0949	0.076	5	1–3
Latissimus dorsi	Superior	201.6	0.2109	0.081	0	1–2
	Middle	315	0.2656	0.095	0	3–4
	Inferior	270.2	0.3062	0.062	0	5–6
Pectoralis major	Clavicle	408.8	0.1087	0.014	0	C1–C2
	Thorax middle	683.2	0.1500	0.026	0	4–6
	Thorax inferior	571.2	0.1830	0.043	0	1–3
Teres major		851.2	0.1410	0.006	0	All
Infraspinatus	Superior	967.4	0.0698	0.050	0	4–6
	Inferior	1,037.4	0.0677	0.084	0	1–3
Pectoralis minor		429.8	0.1183	0.032	0	All
Teres minor		695.8	0.0550	0.051	0	All
Subscapularis	Superior	540.4	0.0676	0.059	5	1–3
	Middle	609	0.0744	0.055	5	4–5, 10
	Inferior	854	0.0721	0.059	0	6–9, 11
Supraspinatus	Anterior	543.2	0.0554	0.031	0	3–4
	Posterior	326.2	0.0591	0.025	0	1–2
Triceps long		1,580.6	0.0969	0.241	10	All
Biceps	Long	485.8	0.1412	0.257	0	All
	Brevis	693	0.1264	0.212	0	All

Reprinted from Muscle contributions to upper-extremity movement and work from a musculoskeletal model of the human shoulder by Seth et al. (17).

The generic model has a default size, which does not yet reflect the actual size of the participant. For both the markerless and marker-based models, it is important that the model is scaled to match the dimensions of each participant (Figures 2 1.D,2.D). For this scaling step, a static measurement of a neutral pose was used. In this neutral pose, scale factors were computed for each body segment, by calculating the factor of the difference in distances between virtual markers on the generic model segments (pink in Figures 2 1.C,2.C) and the corresponding experimental markers (blue and red in Figures 2 1.B,2.B) measured from the participant. This scaling step scales the size and mass properties of the body segments, and many of the elements attached to the segments, including muscle actuators and wrapping objects. Since the camera-based experimental data have only a limited number of markers, the scaling of this model was in some cases less specific than for the marker-based model, meaning that certain scale factors had to be used to scale multiple body segments or directions. For instance, while the front-back width of the thorax can be scaled separately in the marker-based method, the markerless method only has the length (up-down) distance and must use this factor to scale all thorax directions uniformly.

### Inverse kinematics

The scaled models were used for Inverse Kinematics (IK) analysis of each motion trial in OpenSim (Figures 2 1.D,2.D). The IK input data for the marker-based method were different than for the marker-less method, but the procedure was the same for both methods from this step onwards. During IK analysis, the pose of the model that best matched the experimental marker data on each time frame was computed, so the differences between virtual markers on the model and corresponding experimental markers from the participant were minimized. In addition to the input from experimental markers, it is possible to specify certain joint angles that are measured or calculated beforehand as input for IK. In the present study, precalculated joint orientations of the scapula and clavicula [based on regression equations of Pascoal et al. (21)] were used as additional input for IK. The best match of the pose was determined during IK by solving a weighted least squares equation, minimizing both marker errors and joint angle errors between the model and experimental data.

### Static optimization

The IK solution for each motion trial was used as input for Static Optimization (SO) of muscle forces in OpenSim. As additional input, the gravitational force at the dumbbell was applied at the center of mass of the hand as a downwards directed vector of 29.4 N for the lateral fly or 49.1 N for the biceps curl (Figures 2 1.D,2.D). Based on this information, first, net joint moments were estimated through inverse dynamics (ID) for each frame, and subsequently the optimal cost-effective combination of muscle forces was estimated that could have generated this net joint moment, based on minimization of the squared muscle activation (default in OpenSim).

### Data analysis and statistics

For the lateral fly, relevant muscles included all heads of the deltoid and trapezius muscles (Table 2) as these can be considered the prime movers, and the rotator cuff muscles as these are the stabilizing muscles of the shoulder. For the biceps curl, the long and short heads of the biceps were included as these are the prime movers, the triceps muscle was included as this is a potential co-contractor, and the rotator cuff muscles were included as these are the shoulder stabilizers. The time series of muscle forces obtained from the camera-based method were up-sampled to the sample frequency of the marker-based method and synchronized. Synchronization was performed separately for each movement cycle, at the peak shoulder elevation angle for the lateral fly exercise and at the peak elbow flexion angle for the biceps curl exercise. One second before and after the peak instants were included for analysis. Subsequently, muscle forces were filtered with a low-pass 2^nd^ order Butterworth filter with cutoff frequency of 4 Hz. For all repetitions, root-mean-square-deviations (RMSDs) expressed in Newton (N) and in percentage of the maximum force that the muscle can deliver (%Fmax) were calculated between the muscle force time series obtained from the single camera and from the marker-based method. This meant that at each frame, the deviation between the muscle force estimated by the single camera-based and the muscle force estimate by marker-based methods was calculated and squared, the squared deviations were averaged over the whole signal, and the root of this average represented the RMSD for that trial. For each subject, the mean RMSD over all trials for a specific exercise was calculated, and finally, the mean RMSD averaged over all subjects was reported, including a standard deviation that represented the between-subject variation. According to the same method, Pearson's correlation coefficients were calculated per trial and finally averaged over the subjects. The absolute values of *r* were categorized as weak, moderate, strong, and excellent for *r* ≤ 0.35, 0.35 < *r* ≤ 0.67, 0.67 < *r* ≤ 0.90 and 0.90 < *r*, respectively (22). In addition, peak muscle force values estimated by both methods were obtained for the prime movers (that had a large contribution to the total force), and the measurement errors between the two methods were visualized in Bland-Altman plots.

## Result

**Figure 4:**
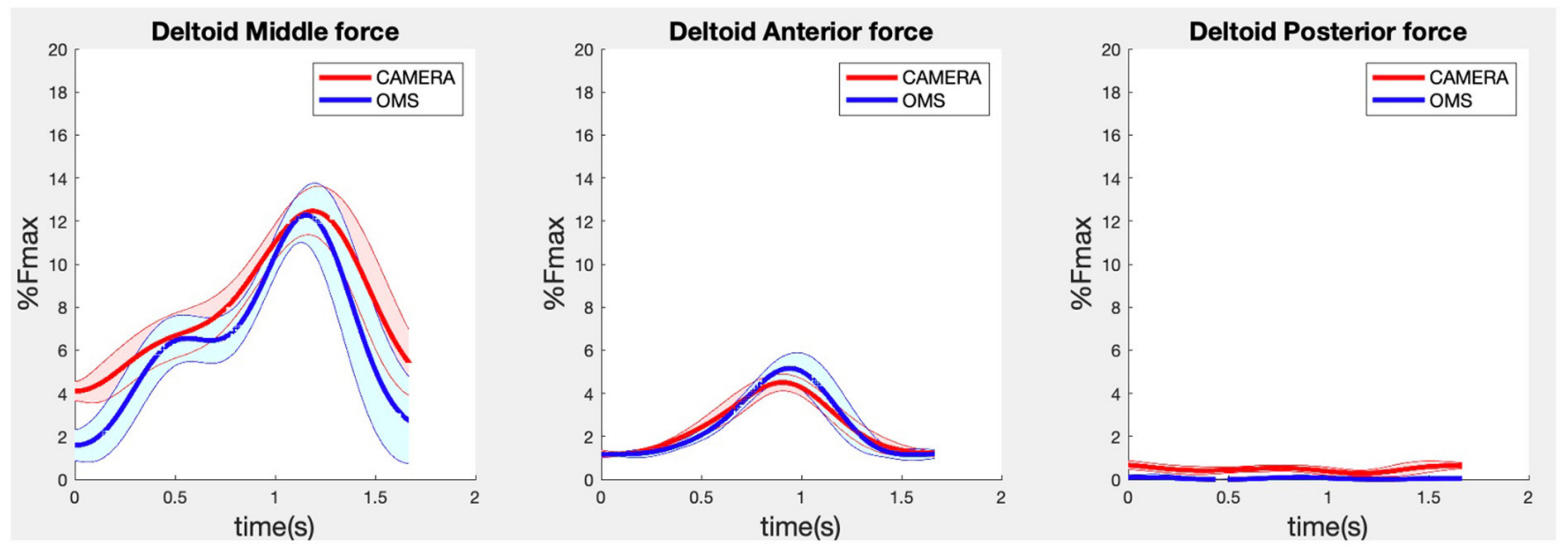
Results for the middle (left), anterior (middle) and posterior (right) deltoid muscle forces during the lateral fly exercise. Results obtained from OMS (blue) and a single camera (red) are presented as mean ± 1SD (shaded) over all participants.

**Figure 5:**
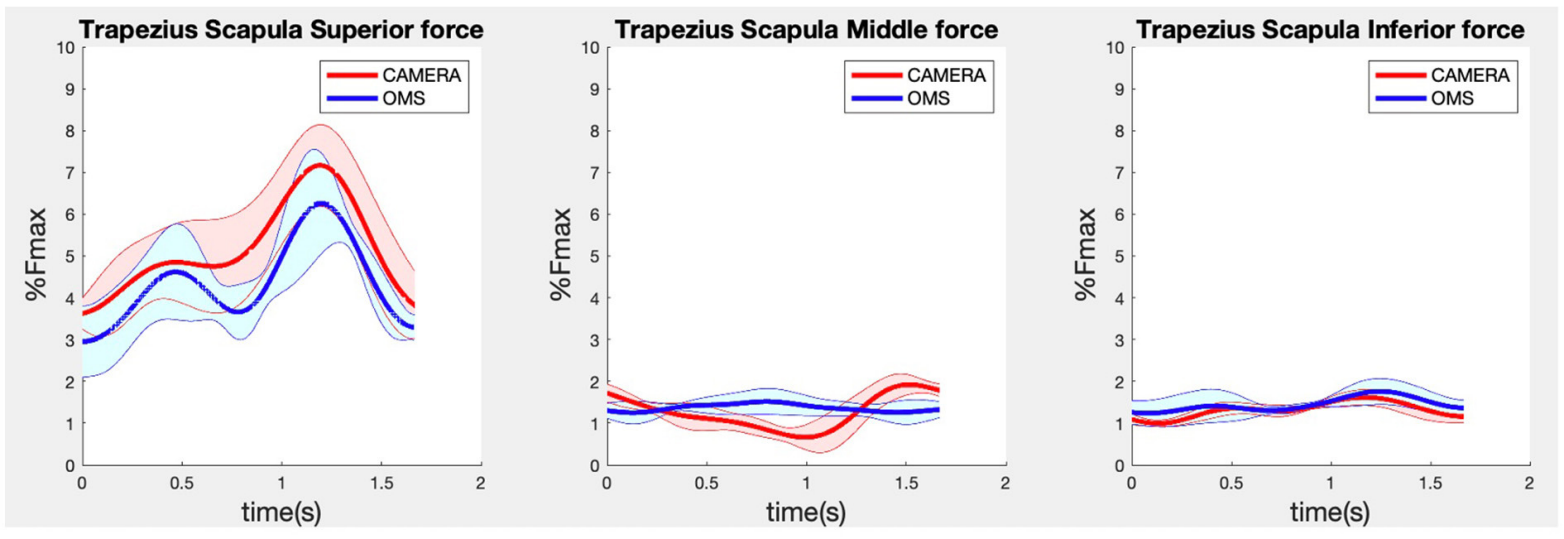
Results for the trapezius scapula superior (left), trapezius scapula middle (middle), and trapezius scapula inferior (right) muscle forces during the lateral fly exercise. Results obtained from OMS (blue) and camera (red) are presented as mean ± 1SD (shaded) over all participants.

**Figure 6:**
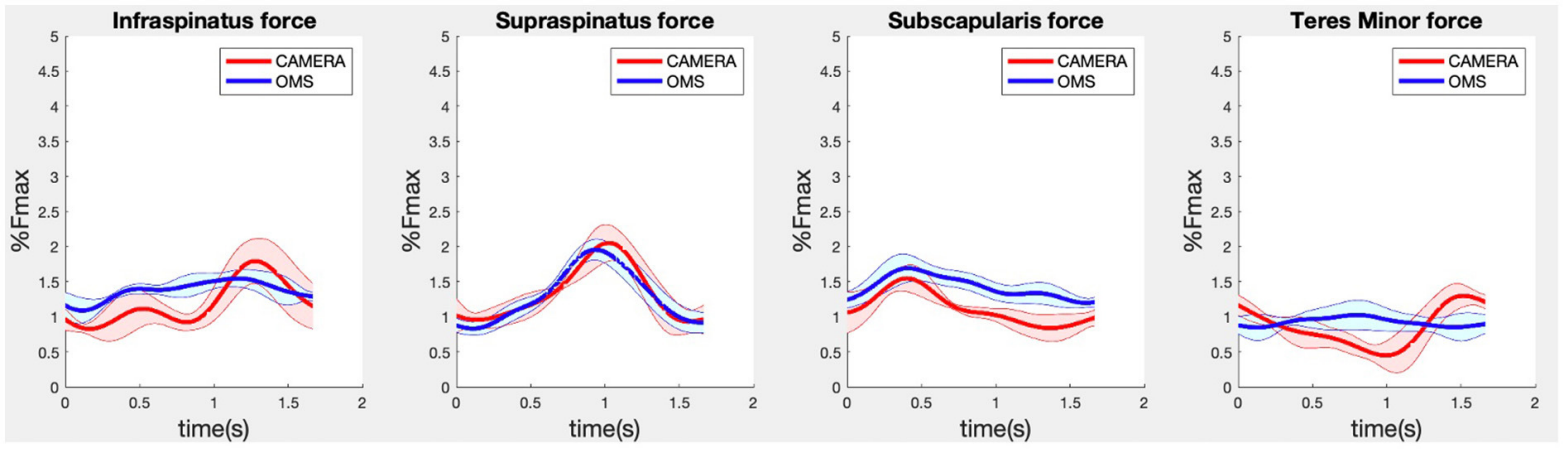
Results for the rotator cuff muscle forces during the lateral fly exercise. Results obtained from OMS (blue) and camera (red) are presented as mean ± 1SD (shaded) over all participants.

**Figure 7:**
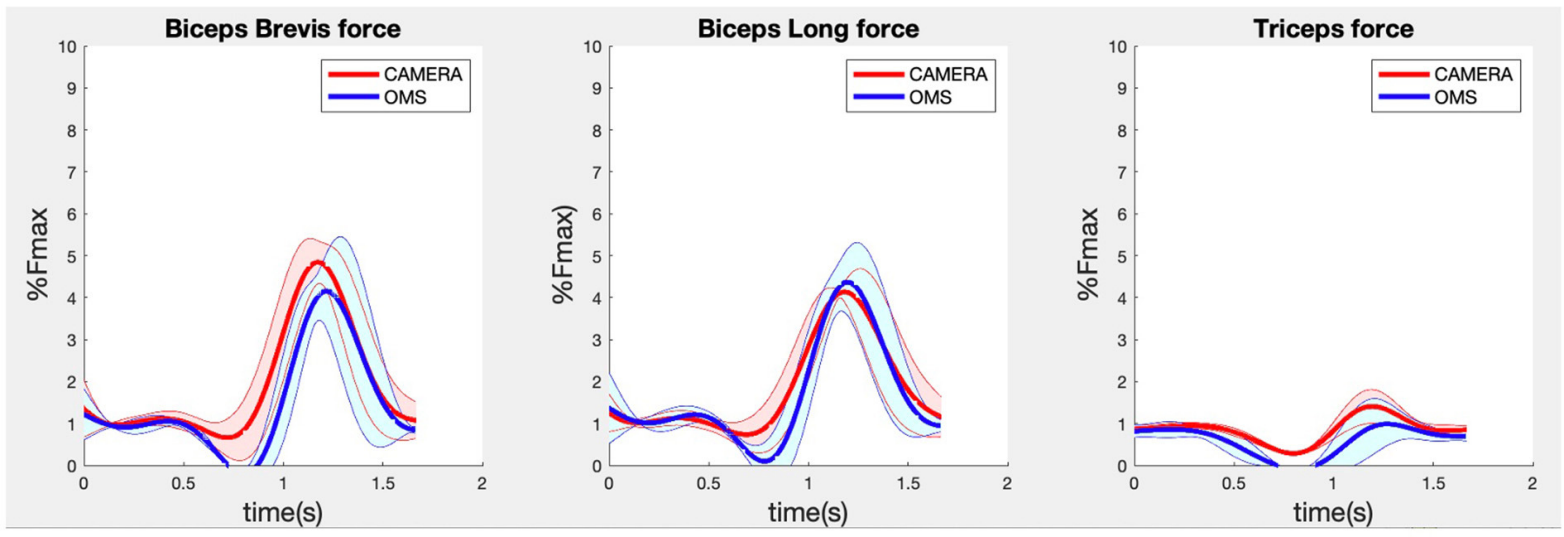
Results for the biceps brevis (left), biceps long (middle) and triceps (right) muscle forces during the biceps curl exercise. Results obtained from OMS (blue) and camera (red) are presented as mean ± 1SD (shaded) over all participants.

**Figure 8:**
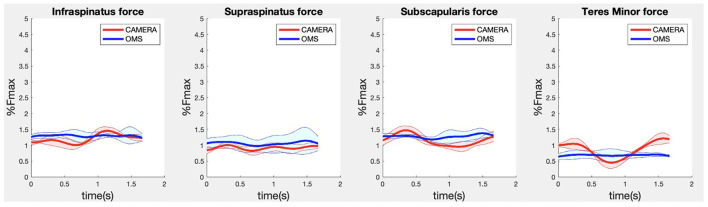
Results for the rotator cuff muscle forces during the biceps curl exercise. Results obtained from OMS (blue) and camera (red) are presented as mean ± 1SD (shaded) over all participants.

Due to missing markers of the OMS, 12% of the lateral fly trials and 23% of the biceps curl trials had to be excluded. Based on the standard camera and deep-learning method, almost all trials could be included except three trials (2%) in which joint estimations were completely insufficient because there was a second person visible in the background. Insufficient trials were identified from the stick figure videos and joint center coordinate data, which for instance showed segment lengths of just a few centimeters, or (unrealistic) stick figure projections at the wrong location not near the participant. In total, 64 lateral fly repetitions and 58 biceps curl repetitions were included for the muscle force analysis. Figures 4–8 show the muscle forces estimated by the two methods during a repetition, averaged first over all trials per subject and subsequently averaged over all subjects. Overall, the magnitude and shape of the muscle forces responsible for the largest part of the force, including the middle deltoid, anterior deltoid, and trapezius scapularis for the lateral fly, and the long and short heads of the biceps for the biceps curl, seemed comparable between the two methods. In line with the figures, correlations were strong or excellent (ranging between 0.71 and 0.92) for these muscles, and RMSDs ranged between 3.1 and 53.3 N, which corresponded to errors of 0.4–2.1% of the maximum force that these muscles can deliver (Table 3, in bold). The other evaluated muscles clearly played a smaller role during the investigated exercises, exerting forces that were typically below 3% of their maximum force, as revealed by Figures 4–8. RMSDs for these muscles ranged between 2.2 and 11.8 N, which corresponded to errors of 0.4–0.7% of the maximum force that these muscles can deliver (Table 3). Correlations for these muscles ranged from weak to strong (Table 3). In addition, it appeared from the Bland-Altman plots (Appendix A) that the camera and deep-learning method tended to underestimate the peak forces of the anterior deltoid and tended to overestimate the peak values of the trapezius scapula superior (during the lateral fly) and biceps brevis (during the biceps curl). Average errors in estimations of middle deltoid and biceps long peak forces were near zero. Moreover, the plots revealed that the between-method errors were usually comparable over the different mean peak force values, meaning that there was no clear sign of proportional errors. However, for both the trapezius scapula superior and biceps brevis peak forces, it was observed that the measurement error was clearly different for one of the subjects (although not for the same subject). These results indicate that the kinematics captured by the camera-based method are not always consistent between different subjects.

**Table 3:** Mean Root-Mean-Square-Deviations (RMSD) expressed in Newton (N) and in percentage of the maximum muscle force (Fmax) and Pearson's correlation (r) between the OMS and the camera methods.

**Muscle**	**RMSD in Newton (mean ± SD)**	**RMSD in % Fmax (mean ± SD)**	**r (mean ± SD)**
**Lateral fly**
Deltoid middle	**53.3** **± 15.6 N**	**2.1** **± 0.6%**	**0.92** **± 0.04**
Deltoid anterior	**5.7** **± 0.6 N**	**0.8** **± 0.1%**	**0.87** **± 0.05**
Deltoid posterior	6.2 ± 1.7 N	0.5 ± 0.1%	0.31 ± 0.21
Trapezius scapula superior	**16.2** **± 2.5 N**	**1.5 ± 0.2%**	**0.72** **± 0.09**
Trapezius scapula middle	3.4 ± 0.6 N	0.7 ± 0.1%	−0.20 ± 0.53
Trapezius scapula inferior	2.2 ± 1.1 N	0.5 ± 0.3%	0.36 ± 0.13
Infraspinatus	11.8 ± 2.1 N	0.6 ± 0.1%	0.30 ± 0.15
Teres minor	3.4 ± 0.6 N	0.5 ± 0.1%	−0.20 ± 0.53
Subscapularis	10.4 ± 1.1 N	0.5 ± 0.1%	0.44 ± 0.17
Supraspinatus	3.5 ± 0.6 N	0.4 ± 0.1%	0.72 ± 0.09
**Biceps curl**			
Biceps brevis	**7.1** **± 0.9 N**	**0.9** **± 0.1%**	**0.84** **± 0.08**
Biceps long	**3.1** **± 0.9 N**	**0.7** **± 0.1%**	**0.90** **± 0.08**
Triceps	7.9 ± 2.8 N	0.5 ± 0.2%	0.61 ± 0.20
Infraspinatus	8.7 ± 2.5 N	0.4 ± 0.1%	0.00 ± 0.23
Teres minor	2.6 ± 0.6 N	0.4 ± 0.1%	0.09 ± 0.43
Subscapularis	7.8 ± 0.8 N	0.4 ± 0.0%	0.19 ± 0.23
Supraspinatus	3.1 ± 0.5 N	0.4 ± 0.1%	−0.04 ± 0.27

RMSDs and correlations were determined over the whole movement cycles. All variables were presented as mean and standard deviation over the averages of participants. Numbers in bold represent results from the muscles that generated the largest parts of the force.

## Discussion

The aim of the present study was to evaluate the feasibility and validity of a single camera CNN driven musculoskeletal model for muscle force estimation during strength exercises. Recordings by this method appeared feasible and fast since only one camera is required. In addition, more trials could be included (fewer missing data), and postprocessing was less effort-consuming since instead of marker cleaning (for a marker-based method) only a pre-made script was used to obtain joint centers from recorded videos. First results regarding the validity showed that the prime movers involved in the upper-body exercises could be estimated by the camera driven musculoskeletal model with reasonable accuracy, indicated by strong to excellent correlations and RMSDs of 0.4–2.1% of the maximum forces. Stabilizing (rotator cuff) and secondary muscles showed similar acceptable RMSDs between 0.4 and 0.7% of the maximum force but correlations that ranged from weak to strong. The low contribution of these muscles makes the validity more difficult to determine, since correlations are sensitive to data distributions and tend to be smaller for variables with a small value range.

The differences compared to the state-of-the art may have multiple explanations. First, the inverse kinematic solution (and therewith muscle force estimation) may have been influenced by the limited number of markers used for the camera-driven model. This limited number of markers may have led to less precise scaling and different information to solve Inverse Kinematics (based on only joint centers instead of many bony landmarks), which could (partially) explain the differences in muscle force estimations. Secondly, muscle force estimation errors may be the result of errors in the kinematics estimated by the deep-learning model, which will likely have had the largest contribution to the total muscle force estimation error. Our previous work has shown that upper-extremity joint angle RMSDs between the deep-learning camera-based and state-of-the-art marker-based biomechanical (linked segment) model, are about 4–8 degrees on average (23), which could also be an explanation for the muscle force estimation errors in the present study. Accuracy might be improved by training the deep-learning model on more and more relevant (strength training) data. In addition, more accurate estimations may be achieved if OpenSim parameters like scaling factors and joint angles, can be estimated directly from videos, without conducting the in-between step of joint center estimation, as fewer steps may reduce the overall error. Furthermore, since the Bland-Altman plots (Appendix A) revealed inconsistencies in the peak force estimations between different subjects, the deep-learning model might be improved by training on more subjects with varying anthropometries.

In comparison with previous work, similar RMSDs and correlations were found for the middle deltoid muscle by Skals et al. (10). They compared the middle deltoid force obtained by a dual depth-sensor driven musculoskeletal model to those obtained by a marker driven musculoskeletal model during loaded and unloaded shoulder elevation and found RMSDs and correlations of 5.45%BW (corresponding to 43 N for a person of 80 kg) and 0.88, and 2.88%BW (corresponding to 23 N for a person of 80 kg) and 0.66, respectively (10). For the deltoid in the present study (Table 3) comparable accuracies were achieved while only the data obtained from a single standard camera were used to drive the musculoskeletal model, whereas Skals et al. (10) used two Kinect depth-sensors. Potentially, this can be explained by the more sophisticated pose estimation algorithm employed in the present study. Whereas, the Kinect extracts pose information based on a random forest approach from single images, the camera-based method used CNNs which can incorporate more temporal and spatial information to optimize predictions (4, 24). In summary, the more feasible camera-driven musculoskeletal model showed comparable accuracies as a dual depth-sensor driven model, although results could only be compared for one muscle.

Multiple limitations applied to the present study. Firstly, the sample size and number of measured exercises was relatively small in the present study, as this was an explorative study into a new technology. It would be interesting for future research to investigate the camera-driven musculoskeletal model in a larger sample and with a larger variety of exercises. Secondly, an opto-electronical measurement system combined with a musculoskeletal model was used as ground truth in the present study, while it is known that soft-tissue artifacts may influence marker measurements, and musculoskeletal model estimations may deviate from true muscle forces because several assumptions regarding muscle parameters, geometries and joint kinematics must be made (25). Nevertheless, this method was the best ground truth option since we were also interested in muscles that could not be measured by surface EMG, and the comparable methodology between the marker-based and camera-based muscle force estimations made it easier to interpret the results as the number of error sources was minimized. Thirdly, the initial idea for this study was to use only marker data (from both systems) as input for IK and no joint angles. However, it appeared that for the marker-based measurements, scapula and clavicula orientations could not be measured accurately with the marker data, probably due to well-developed deltoid muscles of the participants that interfered with the orientation of the cluster marker placed on the acromion, leading to a mismatch between the orientation of the scapula and this cluster marker. Scapula tracking with skin markers remains a large challenge in biomechanical research because the scapula moves beneath the skin and muscles (26). Therefore, regression equations were employed in the present study to estimate scapula and clavicula orientations from the humerus and thorax orientations (21). These estimated coordinates were used as additional input besides the marker data for IK analysis. Since these estimations were added for both the marker-driven and camera-driven models, the error this may have induced will not have affected the comparison. Finally, it must be mentioned that multiple limitations are associated with the single camera-based set up. Single standard cameras can have issues in detecting body segments during occlusions, are known to be sensitive to varying light conditions, and generally provide less accurate results than multi-camera set-ups (6).

The results of the present study may have implications to the strength training science and practice. While it is evident that the validity of the single camera-driven model should be assessed for more subjects and movements, the method shows potential as a feasible tool for movement scientists to investigate (prime mover) muscle contributions during upper-body exercises on a larger scale and in a gym-environment. Information regarding muscle contributions per exercise is currently mainly based on anatomical knowledge, and measurement-based quantification could be valuable to optimize strength training guidelines. In addition, in the future, this method could potentially be applied in the gym to provide strength training athletes with personalized feedback regarding their muscle load during their workout, which could aid in personalized training optimalization and in preventing muscle overload injuries. However, the computation time required by the deep-learning model and OpenSim calculations currently limits the camera-based method to an offline analysis tool, but future research may attempt to apply faster deep-learning and OpenSim models to allow for more direct feedback. The results of the present study may also have implications for other sports. However, as strength training exercises are usually performed at a fixed location, and movements are typically not high-speed, the gym environment seems well-suited for the camera-based method. It would be interesting for future research to investigate if the camera-based method performs similarly for other sports that may deal with high movement speeds or varying distances between the camera and athlete.

In conclusion, a single camera-driven musculoskeletal model is a feasible way to estimate upper-extremity muscle forces during strength exercises. First results regarding the validity show strong to excellent correlations and reasonable RMSDs for prime mover forces compared to those obtained by the state-of-the-art. The accuracy for stabilizing and secondary muscles was difficult to determine due to the low force contributions of these muscles. Future research should validate this method for more subjects and exercises and should focus on further improving the accuracy (e.g., by direct OpenSim parameter estimation from videos). In addition, if the current limitation of computational time could be tackled by future research, this method could be applied in the gym to provide strength training athletes with personalized feedback regarding their muscle load during their workout, which could aid in personalized training directions and preventing muscle overload injuries.

## Data availability statement

The datasets presented in this article are not readily available because videos of participants cannot be shared because of privacy reasons. Requests to access the datasets should be directed to LN, l.noteboom@vu.nl.

## Ethics statement

The studies involving human participants were reviewed and approved by local Ethics Committee of the Faculty of Behavioral and Movement Sciences, Vrije Universiteit Amsterdam. Written informed consent to participate in this study was provided by the participants' legal guardian/next of kin.

## Author contributions

LN: project proposal, data collection, data analysis and statistics, and writing paper. MH: project proposal, assistance in data analysis, assistance in statistics, and reviewing and writing. HV and FV: project proposal, assistance in data analysis, and reviewing and writing. All authors contributed to the article and approved the submitted version.

## Funding

This work was supported by the Dutch Research Council (NWO) under the Citius Altius Sanius Perspective Program P16-28 Project 4.

## Conflict of interest

The authors declare that the research was conducted in the absence of any commercial or financial relationships that could be construed as a potential conflict of interest.

## Publisher's note

All claims expressed in this article are solely those of the authors and do not necessarily represent those of their affiliated organizations, or those of the publisher, the editors and the reviewers. Any product that may be evaluated in this article, or claim that may be made by its manufacturer, is not guaranteed or endorsed by the publisher.
