# Supplementary material for: Feasibility and validity of a single camera CNN driven musculoskeletal model for muscle force estimation during upper extremity strength exercises: Proof-of-concept
Source: Front Sports Act Living. 2022 Sep 23;4:994221. doi: 10.3389/fspor.2022.994221 (PMC9541110; doi:10.3389/fspor.2022.994221)
Supplement: Supplementary file 1 [file Data_Sheet_1.docx]

**Appendix A**. Bland-Altman plots of the peak muscle forces (in Newtons) comparing the estimations from the single camera deep learning-based method and the marker-based method. The Bland-Altman plots can indicate variation in the between-system errors over the different trials, subjects and mean estimated muscle force values. The subplots involve (1) the deltoid middle, (2) the deltoid anterior, (3) the trapezius scapula superior during the **lateral fly** trials, and (4) the biceps brevis, and (5) biceps long during the **biceps curl** trials.


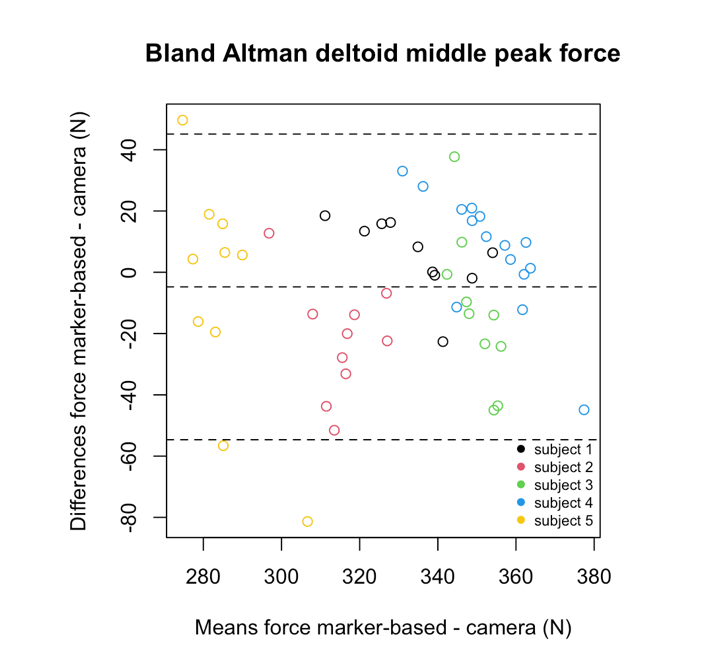


1.


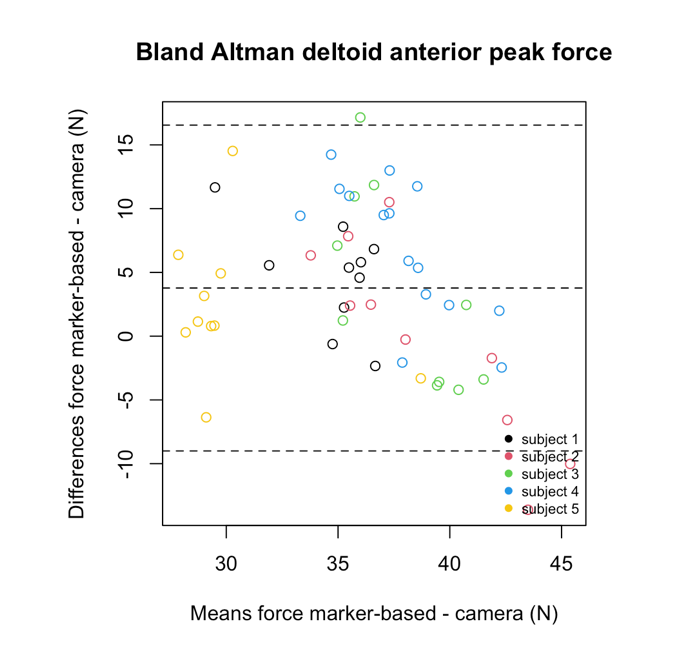


2.


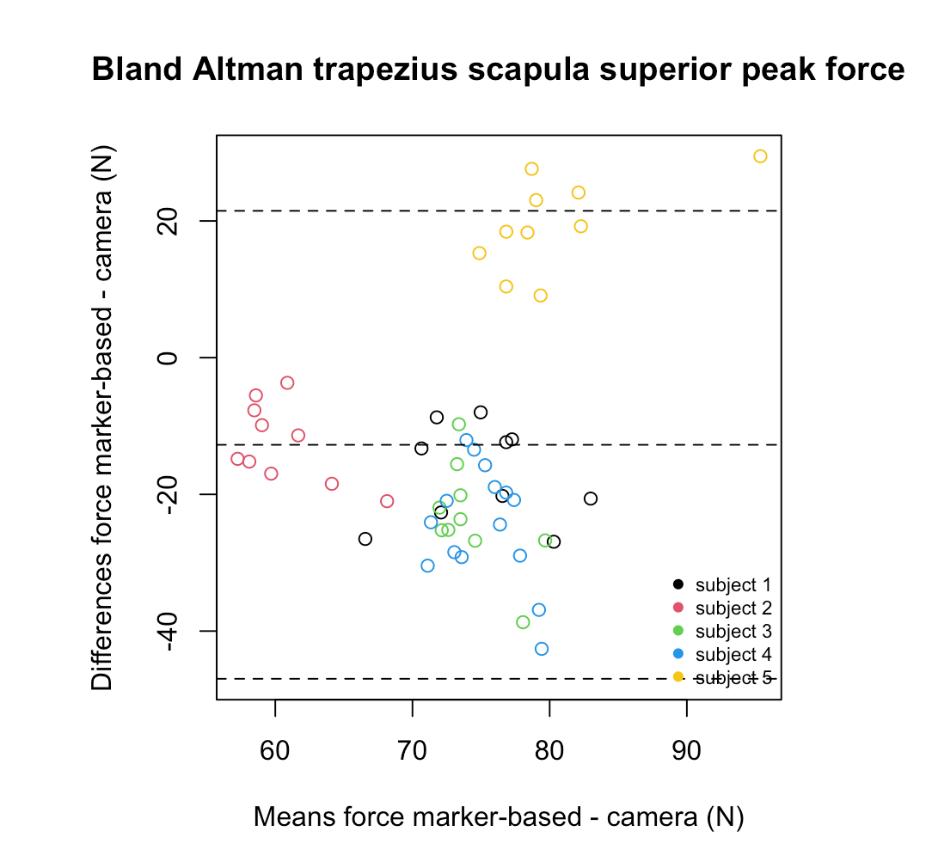
3.

**
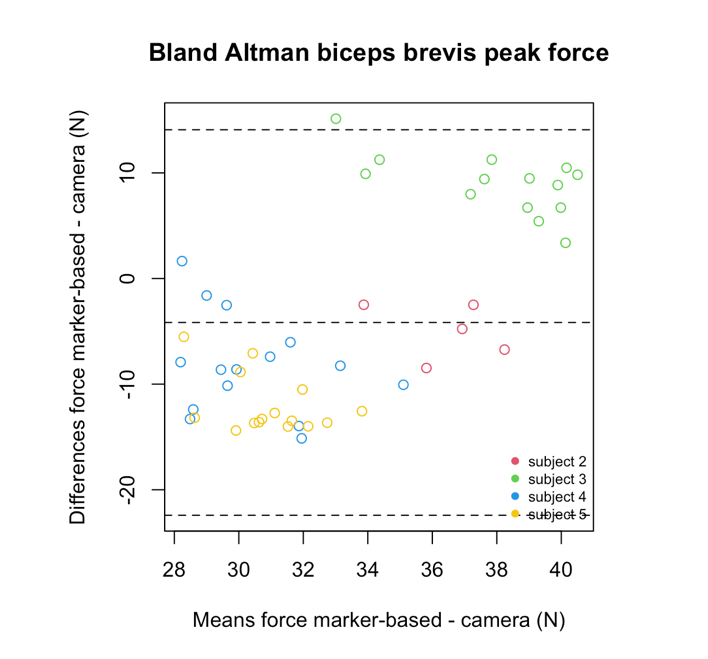
**


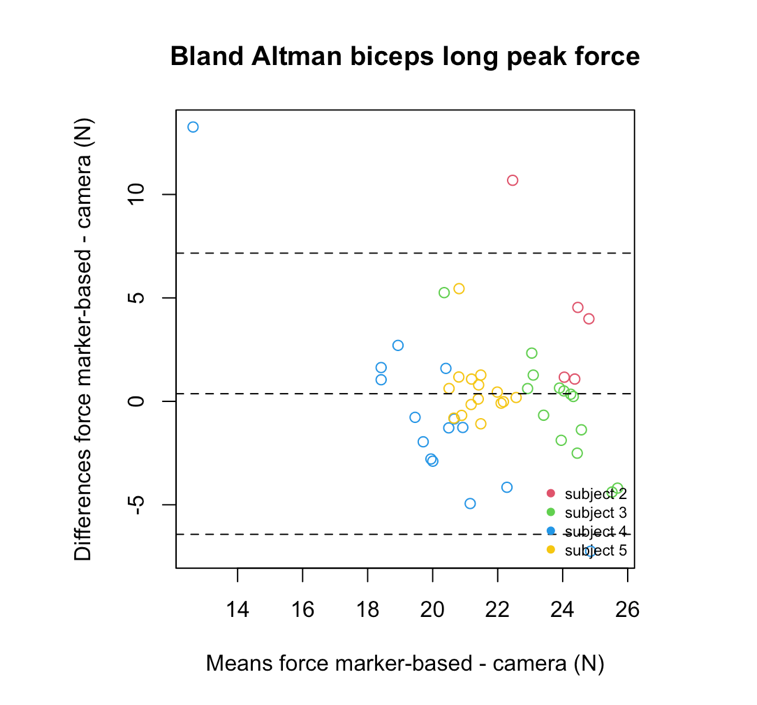
4.


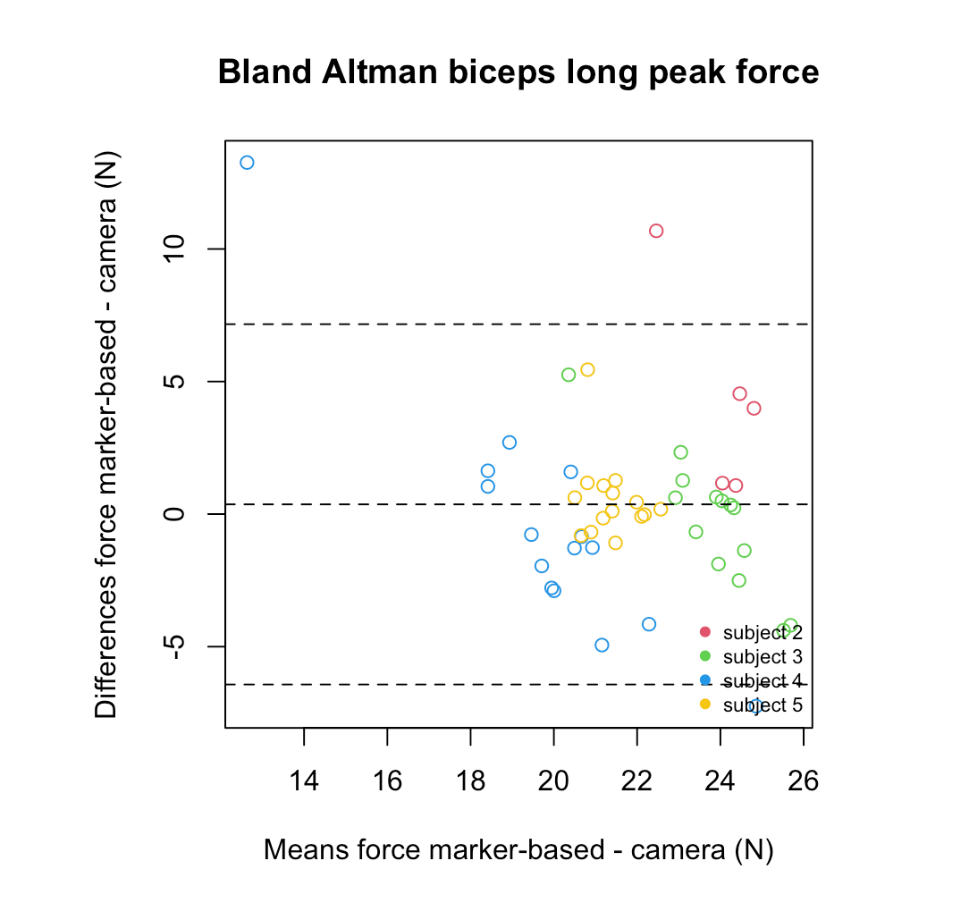


5.
